# Pirfenidone as salvage treatment for refractory bleomycin-induced lung injury: a case report of seminoma

**DOI:** 10.1186/s12885-017-3521-0

**Published:** 2017-08-07

**Authors:** Koji Sakamoto, Satoru Ito, Naozumi Hashimoto, Yoshinori Hasegawa

**Affiliations:** Department of Respiratory Medicine, Nagoya University Graduate School of Medicine, 65 Tsurumai-cho, Showa-ku, Nagoya, 466-8550 Japan

**Keywords:** Bleomycin, Lung toxicity, Pirfenidone, Refractory, Anti-fibrotic agent

## Abstract

**Background:**

Bleomycin-induced lung injury, a major complication of chemotherapy for germ cell tumors, occasionally fails to respond to the standard treatment with corticosteroids and develops into severe respiratory insufficiency. Little is known about salvage treatment for refractory cases.

**Case presentation:**

A 63-year-old man who had been diagnosed with stage I seminoma and undergone a high orchiectomy 1 year previously developed swelling of his left iliac lymph node and was diagnosed with a recurrence of the seminoma. He was administered a standard chemotherapy regimen of cisplatin, etoposide, and bleomycin. At the end of second cycle, he developed a dry cough and fever that was accompanied by newly-identified bilateral infiltrates on chest X-ray. Despite initiation of oral prednisolone, his exertional dyspnea and decline in pulmonary functions continued to be aggravated. High-dose pulse treatment with methylprednisolone was introduced and improved his symptoms and radiologic findings. However, the maintenance dose of oral prednisolone allowed reactivation of the disease with evidence of newly-developed bilateral lung opacities on high-resolution CT scans. Considering his glucose intolerance and cataracts as complications of corticosteroid treatment, administration of pirfenidone was initiated with the patient’s consent. Pirfenidone at 1800 mg/day was well tolerated, and resolved his symptoms and abnormal opacities on a chest CT scan. Subsequently, the dose of prednisolone was gradually tapered without worsening of the disease. At the most recent follow-up, he was still in complete remission of seminoma with a successfully tapered combination dose of prednisolone and pirfenidone.

**Conclusions:**

Pirfenidone, a novel oral agent with anti-inflammatory and -fibrotic properties, should be considered as a salvage drug for refractory cases of bleomycin-induced lung injury.

**Electronic supplementary material:**

The online version of this article (doi:10.1186/s12885-017-3521-0) contains supplementary material, which is available to authorized users.

## Background

Bleomycin is an indispensable antineoplastic agent for the treatment of germ cell tumors and lymphomas. Despite its potent antitumor effect, bleomycin-induced lung injury (BILI) complicates treatment of 7–20% of patients, which often limits its use [1, 2]. Systemic use of corticosteroids is the only standardized therapy for treating BILI. Thus, establishment of an alternative therapy is warranted for cases that have refractory lung injury or cases intolerant of the complications of corticosteroids. Recently, pirfenidone, a novel active small molecule with broad anti-inflammatory and anti-fibrotic potency, has been approved for treatment of idiopathic pulmonary fibrosis [3]. Its potent therapeutic effects on BILI had been observed in the drug development stage using rodent models [4, 5], suggesting its possible application for treatment of BILI in humans. Here we describe a patient with BILI who was successfully improved by pirfenidone after a relapse with systemic corticosteroid treatment.

## Case presentation

A 63-year-old man was referred to Nagoya University Hospital for treatment of a recurrence of resected seminoma. Twelve months prior to his presentation, the patient had undergone high orchiectomy for stage I seminoma (T2N0M0) at a local hospital. Recurrence of the disease was identified by swelling of the left external medial iliac node. After evaluation, he was administered two cycles of chemotherapy with the standard PEB regimen (cisplatin 50 mg/m^2^ on day 1, etoposide 120 mg/m^2^ on days 1 to 3, and bleomycin 30 mg on days 1, 8, and 15). On day 17 of the second cycle, the patient developed fever and malaise. His chest CT scan revealed bilateral ground-glass opacities. On the basis of his clinical picture, BILI was highly suspected. Bleomycin was discontinued, and oral prednisone (30 mg per day) was initiated and improved his symptoms and chest X-ray abnormality immediately. After the completion of two additional courses of chemotherapy without bleomycin, he underwent lymph node dissection. Complete remission was confirmed by pathological examination of the dissected lymph nodes.

**Fig. 1:**
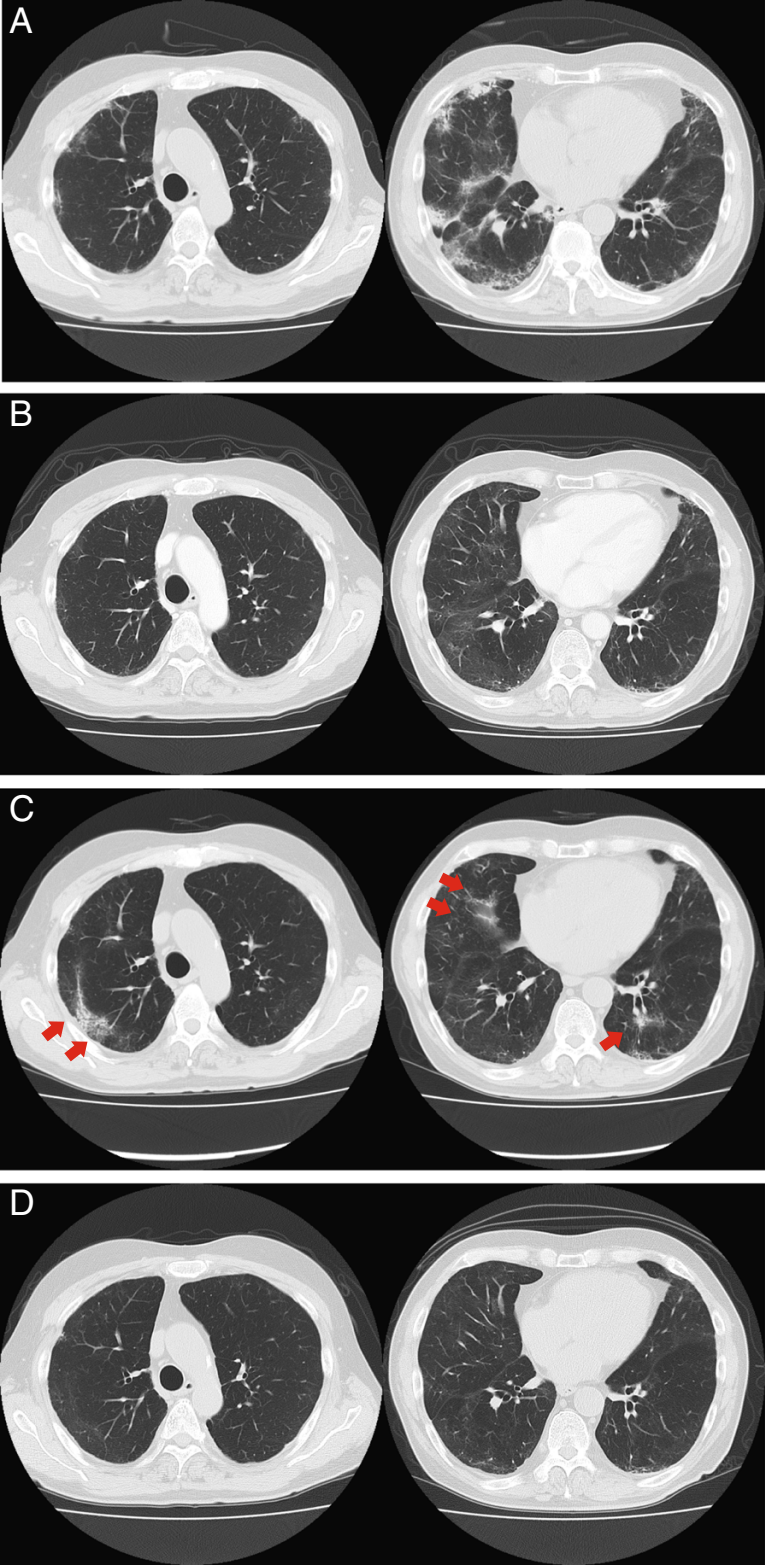
Chest high-resolution CT findings. **a** Ground glass and dense consolidations with patchy distribution were observed in the bilateral lungs before steroid pulse therapy. **b** 3 months after steroid pulse therapy, the bilateral dense consolidation seen in (**a**) was resolved. **c** 8 months after steroid pulse therapy, newly developed bilateral opacities were seen (arrowheads). **d** 1 year after the initiation of pirfenidone add-on therapy

He had suffered from a gradual increase in exertional dyspnea and dry cough since the discharge after the second surgery. For evaluation of his respiratory symptoms, he was admitted to our department. On admission, his physical examination findings were unremarkable except for fine crackles at his bilateral lung bases. The high-resolution CT (HRCT) findings (Fig. 1a) revealed bilateral ground-glass and reticulonodular opacities that had deteriorated compared to the previous study. The pulmonary function test gas was remarkable for marked deterioration of diffusing capacity (Table 1).

**Table 1:** Pulmonary function tests and serum markers

	Before corticosteroid pulse therapy	2 months post corticosteroid pulse therapy	8 months post corticosteroid pulse therapy, before pirfenidone	1 year after starting pirfenidone therapy
Pulmonary Function				
VC (L)	2.60	3.62	3.75	4.12
%VC	78.6	102.4	106.4	103.1
TLC (L)	4.52	5.71	5.53	6.65
%TLC	77.4	97.8	94.5	106.3
DLco(mL/min/mmHg)	11.24	14.70	14.71	15.89
%DLco	56.5	72.8	72.6	78.9
Serum Marker				
KL-6 (U/mL)	472	412	326	287
LDH (IU/L)	328	307	319	237

Bronchoalveolar lavage was performed to exclude other identifiable etiologies, and demonstrated negative results for bacterial culture and an almost normal cell differential count (alveolar macrophages 97%, neutrophils 1%, lymphocytes 1%). Under the diagnosis of exacerbation of BILI, the patient was administered four courses of corticosteroid “pulse” therapy (1 g of methylprednisolone per day, on days 1–3, q7d) to evaluate the maximum effect of corticosteroids. After 1 month of induction therapy, his pulmonary functions and symptoms, as well as HRCT findings, were markedly improved (Fig. 1b). Oral prednisolone (15 mg per day) was introduced as the maintenance dose and gradually tapered to 10 mg per day due to his impaired glucose tolerance.

On the follow-up visit at 8 months after the induction therapy, his HRCT findings demonstrated newly developed ground glass opacities on the outer fields of the bilateral lungs (Fig. 1c). Maintenance therapy with high-dose prednisolone did not seem well adapted to the patient due to his complications (glucose intolerance and cataracts) as well as the insufficiency of its long-term therapeutic effect. With the consent of the patient, administration of pirfenidone was initiated for the non-resolving lung injury with the expectation of sparing the dose of corticosteroids and stabilizing the BILI activity. Pirfenidone at 1800 mg per day orally was well tolerated, and had improved the reticular and ground-glass opacity on HRCT 3 months after initiation. Subsequently, the dose of prednisolone was gradually decreased. One year after the addition of pirfenidone, his HRCT findings (Fig. 1d) and pulmonary function test findings (Table 1) remained improved. At the most recent follow-up, he is still in complete remission for seminoma with a successfully tapered dose of prednisolone (2 mg per day) in combination with the gradually tapering dose of pirfenidone.

## Discussion and Conclusions

Bleomycin is a key drug in induction chemotherapy for malignancies such as Hodgkin lymphoma and germ line tumors [6], but bleomycin-induced lung injury (BILI) is a common complication. In one prospective study in the UK, the incidence of BILI was reported to be 6.8% in patients treated with bleomycin-containing regimens for germ-cell tumors [2].

Risk factors for bleomycin pulmonary toxicity include the cumulative dose of bleomycin, renal insufficiency, smoking history, and use of high partial pressure of oxygen and granulocyte colony stimulating factor. A correlation between the dose and the severity of bleomycin-induced pneumonitis has been found, and it is considered wise to avoid a dose of bleomycin in excess of 400 IU. Nonetheless, cases of BILI with a very low dose use of bleomycin (<50 IU) have also been reported. Patients with BILI present initially with a nonproductive cough, exertional dyspnea, and sometimes fever. These clinical manifestations of BILI are generally encountered weeks to months after the initiation of treatment, but the development of BILI up to 2–10 years after discontinuation of bleomycin therapy has also been reported [7, 8]. Due to the absence of clinical, radiological, or pathologic findings specific to BILI, its diagnosis is generally made by a combination of 1) the exclusion of other etiologies that may cause lung involvement, and 2) radiologic and functional findings compatible with BILI. The former includes negative bacterial cultures of sputum and bronchoalveolar lavage fluids, and the latter includes decreased gas diffusion capacity on lung function tests and bilateral lung opacities in HRCT. Although no controlled trials have been reported, systemic administration of corticosteroids is widely considered to be the standard therapy in symptomatic patients with BILI, but tapering of the corticosteroids sometimes leads to recurrence of clinical symptoms and radiographic findings. Moreover, some insidious cases of lung fibrosis do not respond to corticosteroid therapy [9]. However, an alternative regimen for these refractory and relapsing cases has not been established.

The mechanism of bleomycin-induced lung injury is not entirely clear but likely involves oxidative damage, relative deficiency of the deactivating enzyme bleomycin hydrolase, and amplification of inflammatory cytokines [10, 11]. Bleomycin, an oligopeptide originally isolated from *Streptomyces verticillus,* induces apoptosis in lung epithelia as well as endothelial cells, possibly by causing an oxidant-mediated DNA double-strand break. In the cells, bleomycin is metabolized by bleomycin hydrolase. However, the absence of this enzyme in the lungs has been implicated in the susceptibility to bleomycin toxicity. Damage and activation of these cells as well as alveolar macrophages involved in the inflammation may result in the release of cytokines (IL-1β, TNF-α) and profibrotic growth factors (e.g., TGF-β). This is followed by stimulation of the proliferation and differentiation of myofibroblasts and secretion of a pathologic extracellular matrix, resulting in fibrosis.

Pirfenidone was recently approved as the first line reagent for treating patients with idiopathic pulmonary fibrosis after several prospective large-scale trials confirmed its clinical efficacy for the amelioration of lung fibrosis with well-tolerated adverse event profiles in multiple countries [12, 13]. High oral bioavailability and broad distribution was demonstrated in pharmacokinetic studies [14]. Its potent anti-inflammatory and anti-fibrotic effects were demonstrated in animal models of lung fibrosis including bleomycin-induced lung injury and fibrosis. Oku et al. showed that both prophylactic and therapeutic administration of pirfenidone attenuated lung fibrosis induced by intravenous bleomycin in mice [5]. This was accompanied by the reduction of the proinflammatory and profibrotic mediators that are considered to be key mediators for BILI such as IL-1β, TNF-α, and TGF-β, in the lungs. Of note, a high dose of corticosteroids failed to attenuate lung fibrosis in the same study, suggesting that pirfenidone exerts its therapeutic effect on BILI through distinctive pathways. These findings gave us the idea of managing refractory BILI in this case with pirfenidone.

A group from India recently reported that two cases of BILI successfully recovered after treatment with a combination of pirfenidone, corticosteroid, and N-acetylcysteine [15]. The current case demonstrated marked but transient improvement with high-dose corticosteroid therapy, followed by gradual exacerbation on the maintenance dosage of corticosteroid. Combinatory use of pirfenidone successfully achieved tapering of the corticosteroid dose without worsening lung function. When deterioration of the BILI was observed, we did not increase the dose of corticosteroids, but added pirfenidone instead. Thus, although we cannot exclude the possibility that the clinical resolution was partially due to the concomitant use of steroids, we assume that pirfenidone was mainly responsible for the resolution of recurrent BILI.

In a case of refractory disease with corticosteroid therapy, a successful therapeutic attempt using other reagents targeting molecular pathways involved in BILI such as imatinib has been reported (Additional file 1) [16]. We suggest pirfenidone, a well-tolerated orally-available compound, as another promising candidate for an alternative drug for treating refractory BILI.

## Additional file

Additional file 1: Summary of the treatment experience of BILI with novel therapeutic regimens. PubMed search was conducted in June 2017 with query terms “bleomycin lung toxicity” and “bleomycin lung injury” and limited to clinical studies and case reports, which identified 29 literatures for the recent 15 years. Within them, only 3 reports fulfilled the following criteria; 1) containing treatment information and 2) using pharmacological intervention other than medium-dose corticosteroids for treating BILI. These cases along with our current case were summarized in supplemental table. (PDF 122 kb)

## Abbreviations

BILI: Bleomycin-induced lung injury
HRCT: High-resolution CT
IL: Interleukin
TGF: Transforming growth factor
TNF: Tumor necrosis factor
