# Supplementary material for: Pirfenidone as salvage treatment for refractory bleomycin-induced lung injury: a case report of seminoma
Source: BMC Cancer. 2017 Aug 7;17:526. doi: 10.1186/s12885-017-3521-0 (PMC5547483; doi:10.1186/s12885-017-3521-0)
Supplement: Additional file 1: — Summary of the treatment experience of BILI with novel therapeutic regimens. PubMed search was conducted in June 2017 with query terms “bleomycin lung toxicity” and “bleomycin lung injury” and limited to clinical studies and case reports, which identified 29 literatures for the recent 15 years. Within them, only 3 reports fulfilled the following criteria; 1) containing treatment information and 2) using pharmacological intervention other than medium-dose corticosteroids for treating BILI. These cases along with our current case were summarized in supplemental table. (PDF 122 kb) [file 12885_2017_3521_MOESM1_ESM.pdf]

Supplementary table 1: Summary of the treatment experience of BILI with novel therapeutic regimens

Reference	Type of cancer	Age	Sex	Chemo-therapy regimen	Cycles of chemo-therapy	Novel therapeutics used to ameliorate BILI	Preceding treatment with low dose of steroids	Concomitant use of corticosteroids	Outcome
[16]	Hodgkin's lymphoma	65	male	AVBD	6	**300 mg of imatinib mesylate**	methylprednisolone 2 mg/kg	?, possibly yes	resolution of BILI
[15]	Hodgkin's lymphoma	55	female	unknown	5	**pirfenidone (400 mg tid) and NAC (600 mg tid)**	-	yes, 40 mg per day	resolution of BILI
[15]	Hodgkin's lymphoma	45	male	AVBD	6	**pirfenidone (600 mg tid) and NAC (600 mg tid)**	prednisolone 1 mg/kg	yes, prednisolone 2 mg/kg per day	resolution of BILI
[17]	Hodgkin's lymphoma	65	female	AVBD	4	**pulse corticosteroid (methylprednisolone, 1 g per day)**	methylprednisolone 240 mg per day with NAC	-	marked improvement, some fibrosis remained
Current case	Seminoma	63	male	PEB	2	**pirfenidone (600 mg tid)**	prednisone, 10-30mg per day	yes, prednisone 10mg per day	resolution of BILI

abbreviations: AVBD = adriamycin, vincristine, bleomycin, doxolvicine; PEB = cisplatin, etopoxide, bleomycin; NAC = N-acetylcysteine; BILI = bleomycin-induced lung injury; tid = three times a day

supplementary reference

17. Gupta R, Ettinger NA. Beyond conventional therapy: role of pulse steroids in bleomycin induced lung injury. Respir Care. 2014 Jan;59(1):e9-e12.
